# Efficacy of short-term moderate or high-dose statin therapy for the prevention of contrast-induced nephropathy in high-risk patients with chronic kidney disease: systematic review and meta-analysis

**DOI:** 10.6061/clinics/2021/e1876

**Published:** 2021-03-15

**Authors:** Yan-Lin Zhou, Li-Qun Chen, Xiao-Gang Du

**Affiliations:** Department of Nephrology, The First Affiliated Hospital of Chongqing Medical University, Youyi Road 1, Chongqing 400042, China.

**Keywords:** Contrast-Induced Nephropathy, Contrast-Induced Acute Kidney Injury, Statin, Chronic Kidney Disease, Renal Insufficiency, Diabetes Mellitus, High-Risk Patients

## Abstract

Although previous studies have indicated that statin therapy can effectively prevent the development of CIN, this observation remains controversial, especially in high-risk patients.

A meta-analysis was performed to evaluate the efficacy of statin pretreatment for preventing the development of CIN in patients with chronic kidney disease (CKD) and to determine its effectiveness in various subgroups.

We searched the online databases PubMed, EMBASE, and the Cochrane Library. RCTs that involved the comparison of the short-term moderate or high-dose statin pretreatment with placebo for CIN prevention in CKD patients undergoing angiography were included. The primary outcome was CIN prevalence.

Seven RCTs comprising 4256 participants were investigated in this analysis.

The risk of developing CIN in patients pretreated with statins was significantly lower than that in patients pretreated with placebo (RR=0.57, 95%CI=0.43-0.76, *p*=0.000). The SCr values of the statin group, when analyzed 48h after angiography were lower than those of the placebo group ((SMD=-0.15, 95% CI=-0.27 to -0.04, *p*=0.011). In the subgroup analysis, statin pretreatment could decrease the risk of CIN in CKD patients with DM (RR=0.54, 95% CI=0.39-0.76, *p*=0.000), but not in CKD patients without DM (RR=0.84, 95% CI=0.44-1.60, *p*=0.606). The efficacy of atorvastatin for preventing CIN was consistent with that observed with the use of rosuvastatin. The risk ratios (RR) were 0.51 (95% CI=0.32-0.81, *p*=0.004) and 0.60 (95% CI=0.41-0.88, *p*=0.009), respectively.

Our study demonstrated that statin pretreatment could prevent the development of CIN in CKD patients. However, subgroup analysis demonstrated that statin pretreatment, despite being effective in preventing CIN in patients with CKD and DM, was not helpful for CKD patients without DM. Rosuvastatin and atorvastatin exhibited similar preventive effects with respect to CIN.

## INTRODUCTION

Contrast-induced nephropathy (CIN), or contrast-induced acute kidney injury (CI-AKI), is a common complication associated with angiographic procedures. It is caused by the intravascular administration of contrast medium (CM) injection, and is the third leading cause of hospital-acquired AKI, accounting for 12% of all cases, next to hypoperfusion (42%) and postoperative renal injury (18%) (1). Although the risk of developing CIN has been decreasing in recent years owing to protective measures, the occurrence of CIN still cannot be prevented. Suspected cases of CI-AKI are often associated with particularly high frequency of renal replacement therapy, resulting in significantly increased mortality, extended hospitalization periods, and additional costs (2). This complication is also strongly linked to a greater risk of chronic kidney disease (CKD) development and death, even subsequent atherosclerotic cardiovascular events (3). The occurrence of CIN is found to be associated with multiple risk factors (4), among which CKD and DM are the most dominant. CIN occurs more frequently in patients with CKD and may be exacerbated in patients with diabetic nephropathy (5). The incidence of CI-AKI in CKD patients who underwent standard PCI procedures was reported to be as high as 30.6% (6).

As there is no specific treatment for CIN, it is critical to accurately evaluate the risk factors associated with the occurrence of CIN and to take some measures to prevent the occurrence of CIN in patients at a high risk of developing CI-AKI. Adequate hydration before the procedure and following CM exposure is recommended by guidelines (31)as the principal prophylactic intervention. Despite 90% of the iodinated contrast being eliminated after few hours of hemodialysis, the procedure does not reduce the incidence of CIN (8). Moreover, available data from randomized controlled trials (RCTs) have demonstrated the efficacy of some pharmacological drugs, including N-acetylcysteine (NAC), statin, sodium bicarbonate, and ascorbic acid (9) at preventing the occurrence of CIN. However, no consensus has been reached regarding the optimal or most beneficial treatment for high-risk populations. For example, statin therapy for preventing CIN is still controversial. One study has shown that high-potency statins, including rosuvastatin (≥10 mg/day), atorvastatin (≥20 mg/day), and simvastatin (≥40 mg/day), may be associated with an increased incidence of AKI (10). However, most studies indicate that statins have a renal-protection effect in the context of CIN. Most of the included subjects were the patients along with normal kidney function, and few studies have been performed in high-risk patients with CKD (32,33). For example, a meta analysis (11) including 5174 patients demonstrated that high-dose statin therapy is effective at preventing the development of CIN in patients undergoing PCI for acute coronary syndromes (ACS). Therefore, we subjected RCTs to a meta-analysis to further evaluate the efficacy of short-term moderate or high-dose statin pretreatment at preventing the occurrence of CIN in high-risk patients with CKD, in addition to ascertaining its effectiveness in various subgroups.

## METHODS

### Search strategy

An extensive literature search was performed using three computerized databases: PubMed, EMBASE, and the Cochrane Library from January 1990 to January 2019. We searched each database for studies published in English using search terms (“statin” OR “simvastatin” OR “rosuvastatin” OR “atorvastatin” OR “pravastatin” OR “fluvastatin”) AND (“high-risk patients” OR “CKD” OR “chronic kidney disease” OR “renal insufficiency”) AND (“CIN” OR “contrast-induced nephropathy” OR “contrast-induced acute kidney injury”). In turn, the references sections of the selected articles were reviewed for other potentially relevant citations. Finally, the authors of the selected studies were personally contacted to obtain further information.

### Study selection

**Figure 1:**
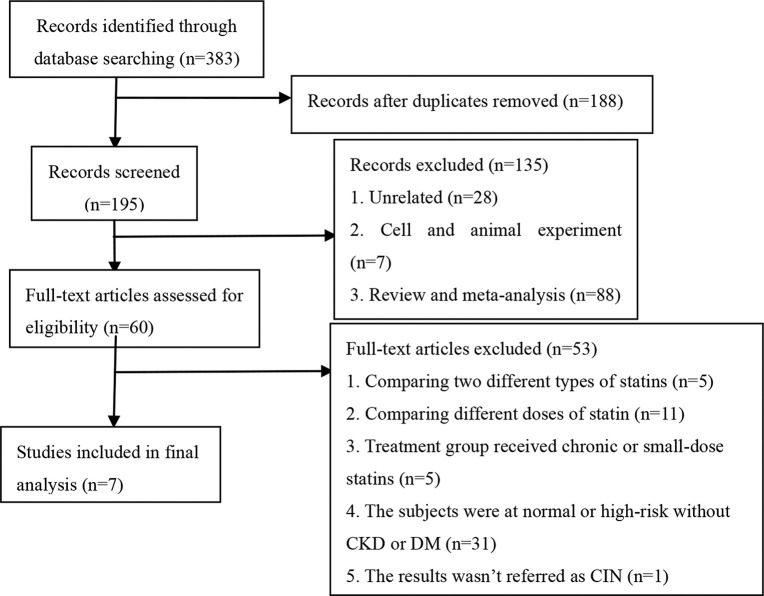
Flow diagram depicting the workflow used for study selection.

The following were the criteria for including studies in this analysis: 1) the study was a randomized controlled trial investigating the efficacy of statins at preventing CIN; 2) the subjects were high-risk patients with CKD; 3) compared with the placebo group, the treatment groups received short-term moderate or high-dose statins at any time before the contrast exposure, wherein moderate/high-dose statins were defined in the following manner: rosuvastatin ≥10 mg/day, atorvastatin ≥20 mg/day, and simvastatin ≥20 mg/day; 4) all participants were injected with an iodine contrast agent intravenously or intra-arterially; 5) the follow-up time was at least 24h; 6) the outcome was CIN. The following exclusion criteria were employed for the studies: 1) reviews, meta-analyses, non-clinical trials such as animal trials, duplicated publications, case reports and letters; 2) non-RCTs; 3) the subjects were normal or high-risk patients without CKD; 4) the trials involved direct comparisons between two different doses or types of statins, or the treatment group received long-term or small-dose statins; 4) the sample size was less than 50 or the follow-up time was less than 24h; 5) the results were associated with the above outcome or they were incomplete; 6) the full-text article was unavailable. Two investigators assessed all articles generated for relevance independently. First, all identified titles and abstracts were reviewed. Second, articles with full text were included/ excluded according to inclusion/exclusion criteria after performing a full-text review. When the eligibility was uncertain, a third reviewer resolved any disagreements between the other two reviewers through discussion. A flowchart depicting the search strategy is provided (Figure 1).

### Data Extraction

Two investigators (YLZ and XGD) extracted all the data independently and achieved consensus regarding all relevant items. The following information was extracted from each study: 1) name of the first author, 2) year of publication, 3) study nation, 4) study design, 5) patient characteristics (number, mean age, male proportion, baseline SCr values, postprocedural change in SCr), type of contrast media, statin type and dose, treatment of control group, specific definition of CIN, and clinical outcomes. In case of disagreements, a third reviewer (LQC) cast the deciding vote.

### Quality assessment

The methodological quality of identified studies was independently evaluated by two reviewers (YLZ and XGD), and disagreements were resolved by consensus and adjudicated by a third reviewer (LQC). We employed the Jadad scoring system to assess study quality. The quality assessments involved concealment of treatment allocation, similarity of the study groups at baseline, eligible criteria, use of any blinding procedure, completeness of the follow-up study, and intention-to-treat analysis. Each article was assigned a quality score based on the following criteria: 1) the grouping was randomized; if the sequence was generated by computer or a random number table, 2 points were given; if the experiment did not explain the method, 1 point was given, otherwise no point was given; 2) a double-blind design was employed; if the process of implementing a double-blind method was detailed, 2 points were given; if the double-blind method was merely mentioned but not described in detail, 1 point was given, otherwise no point was given; 3) if data on lost follow-up were described in detail, 1 point was given, otherwise no point was given. The score range was 0-5 points, wherein a score of 1-2 represented low-quality literature, and a score of 3-5 represented high-quality literature.

### Statistical analysis

All statistical analyses in this meta-analysis were performed using Stata, version 12.0 (StataCorp LP). First, Cochran’s Q statistic test was used to estimate heterogeneity between studies, which was quantified using the I^2^ statistic. We considered I^2^ less than 25%, within 25-50%, and greater than 50% as low, moderate, and high amounts of heterogeneity, respectively. When I^2^ was greater than 50%, a random-effect model was used. Otherwise, a fixed-effect model was employed. Dichotomous data, obtained by calculating the number of high-risk CKD patients who presented CIN, were expressed as risk ratio (RR) with 95% confidence interval (CI) to evaluate the effect of statins. Continuous data pertaining to SCr values were shown as standardized mean difference (SMD) with 95% CI. To further identify potential differences in treatment across the studies, subgroup analyses were conducted on CKD patients with or without DM based on different types of statins used. All the tests were two-tailed and *p*<0.05 was considered significant in this meta-analysis.

## RESULTS

### Characteristics of the studies included in this meta-analysis

After a comprehensive search, 383 potentially relevant articles were screened, of which, 376 were eliminated from the analysis for multiple reasons (Figure 1). Finally, seven studies (12-18) involving 4256 CKD participants were included in our meta-analysis aimed at evaluating the effect of statins on CIN (placebo was used as control). Characteristics of participants and studies are described in Table 1. All of the included studies were RCTs, published in English between 2008 to 2018. Peripheral vascular examination, coronary angiography, and PCI were performed by arterial injection of nonionic osmotic agents. The mean age of all the participants ranged from 51.87±8.48 to 76±7 years, and the percentage of men varied across studies (range, 53-73.4%). The mean baseline SCr ranged from 1.074±0.236 mg/dl to 2.002±0.396 mg/dl. Four studies (13-15,18) evaluated the effect of atorvastatin, two studies (16,17) evaluated the effect of rosuvastatin, and one study (12) evaluated the effect of simvastatin. The criteria used to define CIN were similar among studies with an increase ≥25% from baseline or an absolute increase in Scr ≥0.5 mg/dl and the observation time ranged from 24h to 5d.

### Assessment of the study quality and publication bias

A quality assessment of the included studies is summarized in Table 2. The included studies provided detailed information regarding the eligible criteria and the completeness of the follow-up. Further, all the patients exhibited similar baseline characteristics. Of the seven studies, six involved appropriate randomization methods, five reported blinding of both patients and care providers to treatment assignment, four involved concealment of allocation, and three studies included the intention-to-analysis. Only one study did not provide details that would have enabled the assessment of the appropriateness of randomization.

### Study outcomes

#### Incidence of CIN

**Figure 2:**
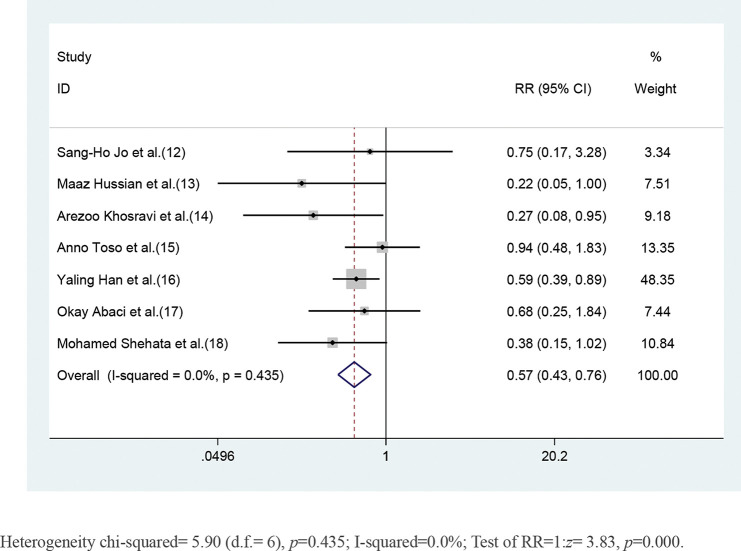
Forest plot depicting risk ratios with 95% CI for the incidence of CIN among high-risk patients with CKD administered statins *versus* control. The blue square on the left/right or in the middle of the line favors statins group/control group or does not favor either of them.

CKD patients who received short-term moderate or high-dose statin pretreatment were at 43% lower risk of developing CIN compared with those in the placebo group based on a fixed-effect model (RR=0.57, 95% CI=0.43-0.76, *p*=0.000). No significant heterogeneity was identified across studies (I^2^=0, *p*=0.453) (Figure 2).

### Parameter changes in SCr

**Figure 3:**
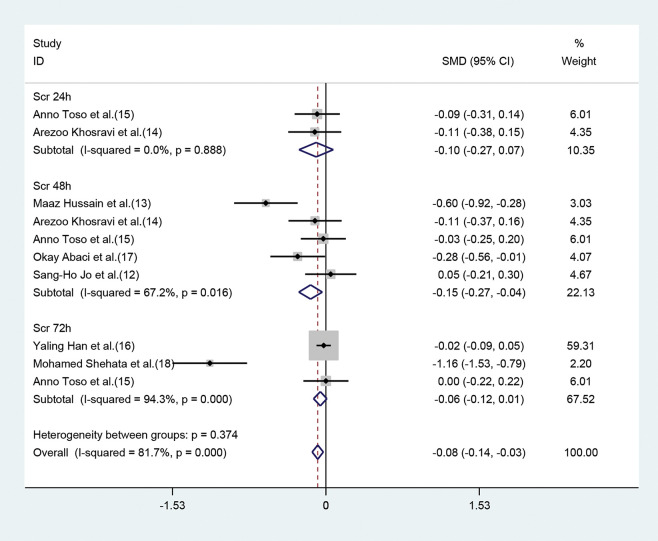
Comparison of SCr values between the statin group and control group at 24, 48 and 72h. The blue square on the left/right or in the middle of the line favors statins group/control group or does not favor either of them.

Of the seven studies, five (12-15,17) exhibited a significant decrease of SCr values 48h post-operation (SMD=-0.15, 95% CI=-0.27 to -0.04, *p*=0.011) (Figure 3), in the statin group (compared with the placebo group). Although the parameter was based on the random-effect model, heterogeneity still existed (I^2^=67.2%). However, significant differences were not observed between moderate or high-dose statin group and the control group in terms of SCr values (SMD=-0.10, 95% CI=-0.27 to 0.07, *p*=0.265) (Figure 3) 24h after the operation in two studies (14,15), and 72h after the operation (SMD=-0.06, 95% CI=-0.12 to 0.01, *p*=0.097) in three studies (15-17). The heterogeneity of the three studies (15-17) was high even upon employing the random method (I^2^=94.3%, *p*=0.0000) (Figure 3).

### Subgroup analysis

#### Effect of different statins on the CIN in high-risk patients with CKD

**Figure 4:**
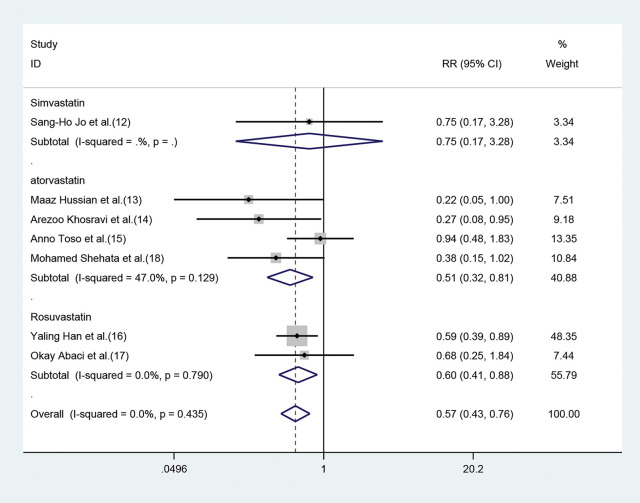
Forest plot depicting subgroup analysis of RR and 95% CI for CIN among high-risk patients with CKD assigned to different statin treatments *versus* placebo. The blue square on the left/right or in the middle of the line favors statins group/control group or does not favor either of them.

Both atorvastatin and rosuvastatin could effectively reduce the risk of CIN development in patients with CKD, and the RRs were 0.51 (95% CI=0.32-0.81, *p*=0.004) and 0.60 (95% CI=0.41-0.88, *p*=0.009), respectively (Figure 4). Only one study was based on simvastatin; however, no CIN prevention effect was found in patients with baseline renal insufficiency undergoing angiography after pretreatment with a high dose of simvastatin for the short term (RR=0.75, 95% CI=0.17-3.28, *p*=0.702).

#### Effect of statins on CIN in CKD patients with or without DM

**Figure 5:**
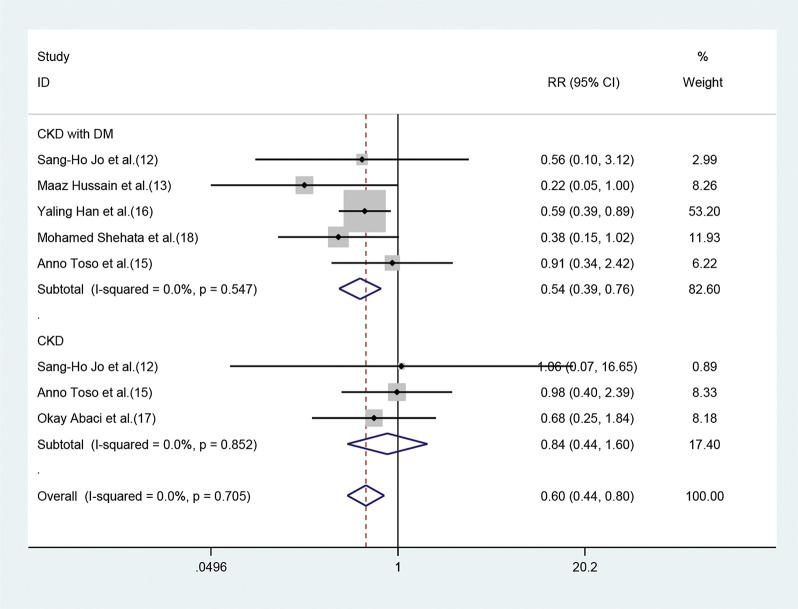
Forest plot depicting subgroup analysis of RR and 95% CI among CKD patients with or without DM assigned to statins *versus* control. The blue square on the left/right or in the middle of the line favors statins group/control group or does not favor either of them.

Of the seven studies, five involved 3411 patients who had renal insufficiency with DM. The fixed-effect model revealed that short-term moderate or high-dose statin pretreatment could effectively prevent the occurrence of CIN (RR=0.54, 95% CI=0.39-0.76, *p*=0.000). In the three studies focused on CKD patients without DM, a significant difference was not observed between the statin and placebo groups (RR=0.84, 95% CI=0.44-1.60, *p*=0.606) (Figure 5).

## DISCUSSION

The present meta- analysis indicated that treatment with short-term moderate or high-dose statin during the periprocedural time of angiography is strongly associated with a significantly lower incidence of CIN in high-risk patients with preexisting CKD. Although the pathophysiological mechanisms underlying CIN have not been fully elucidated, it is generally believed that renal medulla ischemia-hypoxia, oxidative stress injury, inflammation, and direct tubular toxicity of CM are related to the pathogenesis and progression of CIN. In response to stimulation with contrast agents, endothelial cells may release more endothelin and adenosine, and by extension, the expression of nitric oxide (NO), prostaglandin (PGE2), and other vasodilator factors is decreased, resulting in renal ischemia, hypoxia, and acute tubular necrosis. The osmotic load of CM enhances the interstitial pressure and sodium transport, thereby resulting in oxygen consumption. The intense vasoconstriction and loss of autoregulatory capacity could accelerate kidney damage under the influence of reactive oxygen species (ROS) (19). Renal tissues affected by high osmotic load could make intense focal or diffuse vacuolization of the proximal tubules or overt tubular necrosis appear (20). Statins attenuate CIN through modulation of inflammation and endothelial function, besides reducing oxidative stress and apoptosis (21-23).

At present, many clinical trials have been designed to assess the effect of short-term moderate or high-dose statin treatment on CIN, but the efficacy of such a treatment remains controversial. A meta-analysis by Liu et al. (24), which included 9 RCTs involving a total of 5143 patients, demonstrated that preprocedural statin treatment could reduce the risk of developing CIN (RR=0.47, 95% CI=0.37-0.60, *p*<0.0001). Moreover, statin therapy was found to be effective at reducing the incidence of CIN in high-risk patients with preexisting renal dysfunction or DM in the subgroup analysis. These findings were similar to those of another meta-analysis (25). The meta analysis included nine randomised controlled trials with a total of 5143 patients. In the subgroup analysis with only 1330 patients with preexisting renal impairment defined as GFR <60 mL/min/1.73 m2 or creatinine clearance <60 mL/min and excluding patients on dialysis, statin reduced CIN risk by 54% (MH-RR=0.46, 95% CI 0.29 to 0.72, *p*=0.0008). It can be seen that this previous meta-analysis—based on the hypothesis that statins play an important role in preventing the development of CIN— included all subjects. The patients included in our meta-analysis were at high risk of developing CKD and the results showed that statins exhibited a preventive effect against CIN. To our knowledge, our meta-analysis might be the first to focus on patients at a high risk of developing CKD and to explore the preventive effect of moderate or high-dose statins pretreatment on CIN.

Furthermore, our subgroup analysis revealed that statins could effectively reduce the occurrence of CIN in patients with CKD who were also diagnosed with diabetes, but these statins did not exhibit a protective effect against CIN in CKD patients without DM. As is well-known, oxidative stress is independently associated with the pathogenesis of diabetic nephropathy (DN). Prolonged hyperglycemia, accumulation of advanced glycation end products, and increased levels of activated transforming growth factor (TGF)-b1 in the glomerular and tubular epithelial cells can result in increased production of ROS, which contribute to oxidative stress. Therefore, protecting renal cells by suppressing oxidative stress is believed to be a potential therapeutic strategy for DN (26). Abe’s et al. (7) found that treatment with rosuvastatin might decrease the levels of urinary 8-hydroxydeoxyguanosine (8-OHdG), a sensitive indicator of oxidative DNA damage that correlates significantly with the severity of tubulointerstitial lesions**.** In summary, analysis of the studies included in this meta-analysis revealed that rosuvastatin can ameliorate systemic oxidative-stress- and anti-inflammatory reduction-induced tubulointerstitial lesions in DN and. However, no preventive effect was observed in CKD patients without DM. In fact, there were only 3 studies (12,15,17) that included 625 CKD patients without diabetes, and therefore the small sample size may result in a bias. However, as patients in all the studies included in this analysis were at different stages of CKD and exhibited different SCr baseline values, the exact stage of CKD at which statins will exhibit optimal efficacy is unclear.

Pleiotropic effects varied among different statins. Our meta-analysis demonstrated that atorvastatin and rosuvastatin were more effective at protecting high-risk patients from CIN development. Jo et al. (12) demonstrated that treatment with simvastatin did not result in a significantly decreased risk of developing CIN. Hence, more RCTs using simvastatin are needed to investigate the effect of this molecule on CIN. A meta-analysis on the effects of rosuvastatin, which involved the analysis of 15 RCTs—including a total of 2673 patients (27)—indicated that patients who underwent pretreatment with moderate or high-dose rosuvastatin were at a 55% lower risk of developing CIN compared with those in the low-dose rosuvastatin pretreatment or placebo groups (RR=0.45, 95% CI=0.35-0.58, *p*<0.0001). Another meta-analysis (28), which involved the analysis of nine RCTs—a total of 2200 patients—for evaluating the effect of atorvastatin, revealed that atorvastatin pretreatment significantly decreased the incidence of CIN in patients undergoing coronary angiography (OR=0.46, 95% CI=0.27-0.79, *p*=0.004). A prospective single-blind RCT (29) compared the effectiveness of rosuvastatin and atorvastatin with respect to protecting against CIN in patients with myocardial infarction who are undergoing PCI, and demonstrated that rosuvastatin or atorvastatin exhibited similar efficacy with respect to protecting against the occurrence of CIN. An animal study also demonstrated that atorvastatin significantly inhibited NO system dysfunction and apoptosis, while rosuvastatin effectively inhibited inflammation (30).

During the development of CIN, SCr levels typically begin to increase 24-48h post-angiography, and peak after 3-5d. SCr levels are then restored in majority of patients and reach the baseline within 1-4 weeks. Our meta-analysis revealed that the SCr values of CKD patients in the statin-treatment group were significantly lower than those in the control group 48h after the administration of contrast agent. This finding may suggest that CIN is most likely to occur 48h post-angiography, and SCr levels should be closely monitored. In addition, the SCr levels at 48h and 72h after surgery were analyzed based on random-effect model, and the heterogeneity was still high (I^2^>50%). Possible reasons include the fact that the patients included in all studies were at different stages of CKD and the baseline of SCr levels varied extensively.

This meta-analysis had several limitations. First, only seven studies were included and thus the sample size was not sufficiently large. Second, the CKD patients investigated in this analysis were at stage 1 to 4 of CKD; however, we could not to analyze each CKD stage separately. Third, when analyzing continuous variable (SCr), I^2^ was found to be much greater than 50%, which might affect the efficacy of statins. Fourth, the follow-up outcomes of the included studies did not include patients with CIN requiring dialysis, or cases of death, or side effects associated with statin use such as impairment of liver function and rhabdomyolysis. Fifth, all patients investigated in this analysis had been administered an arterial injection of nonionic osmotic agents, and further studies are warranted to evaluate the efficacy of statins in patients who received an intravenous injection of CM. Finally, neutral or negative studies may not be published in a peer-reviewed journal, while positive studies are more likely to be published, and thus the veracity of the current results might be affected.

## CONCLUSION

Our meta-analysis indicated that the short-term moderate or high-dose statin pretreatment before angiography could reduce the risk of developing CIN in high-risk patients with CKD. Subgroup analysis revealed that statin pretreatment exhibited a preventive effect against CIN in patients with CKD and DM, but not in the CKD patients without DM. Both rosuvastatin and atorvastatin exhibited consistent preventive effects against CIN. However, further studies should be designed to confirm the stage of CKD at which statin pretreatment exhibits optimal efficacy and to confirm the effect of statins on CIN development in DM patients with normal renal function.

## AUTHOR CONTRIBUTIONS

Zhou YL and Du XG extracted all the data independently and achieved consensus on all relevant items. In case of disagreements, a third reviewer (Chen LQ) cast the deciding vote. Two reviewers independently (Zhou YL and Du XG) assessed the methodological quality of the identified studies. Disagreements were resolved by discussion and adjudicated by a third reviewer (Chen LQ). All of the three reviewers completed data extraction and analysis, followed by the meta-analysis.

## Figures and Tables

**Table 1:** Characteristics of patients and interventions of included studies.

No	Author(y)	Nation	Study	Inclusion criteria	Statin group	Control group	Patients NO. (E/C)	
1	Sang-Ho Joet al. (12)	Korea	a prospective, randomized, double-blind, placebo-controlled, 2-center trial	patients with renal insufficiency (15<eGFR≤60ml/min.m^2^ or SCr ≥1.1dl/ml)	Simvastatin (160 mg total, 40 mg orally every 12 hours) and hydration	hydration	118	118
2	Maaz Hussian et al. (13)	India	a prospective,group randomized, double-blind, parallel placebo-controlled, two-arm trial	patients with SCr 1-1.5mg/dl or 60<eGFR<90ml/min.m^2^	Atorvastatin (400 mg total, 80 mg/day)+NAC+hydration	NAC+hydration	80	80
3	Arezoo Khosravi et al. (14)	Iran	a double-blind, placebo-controlledclinical trial	patients with CKD(15<eGFR<60ml/min.m^2^, SCr>1.5mg/dl)or diabetes mellitus	Atorvastatin (160 mg total, 80 mg/day)+NAC+hydration	NAC+hydration	110	110
4	Anno Toso et al. (15)	Italy	a prospective, randomized, placebo-controlled trial	patients with CKD(15<CrCl<90ml/min)	Atorvastatin (32 0mg total, 80 mg/day)+NAC+hydration	NAC+hydration	152	152
5	Yaling Hanet al. (16)	China	a prospective, randomized, controlled, multicenter clinical trial	patients with type 2 DM and concomitant stage 2 or 3 CKD (30<eGFR<90ml/min.m^2^)	Rosuvastatin (50 mg total, 10 mg/day)+hydration	hydration	1498	1500
6	Okay Abaci et al. (17)	Turkey	a randomized controlled trial	Patients with CKD(30<eGFR<60 ml/min.m^2^)	Rosuvastatin (80 mg total, 40 mg/day, then 20 mg/day) +hydration	hydration	103	105
7	Mohamed Shehata et al. (18)	Egypt	a prospective, randomized, double-blind, placebo-controlled trial	diabetic patients with mild to moderate CKD(30<eGFR<90ml/min.m^2^)	Atorvastatin(160 mg total,80 mg/day)+NAC+hydration	NAC+hydration	65	65

Mean age, (years), E/C		Male No (%) E/C	Mean baseline (SCr)E/C(mg/dl)	Contrast medium		Definition of CIN		CIN E/C
65.0±9.3/66.1±8.2		91 (73.4%)/88 ((71.5%)	1.286±0.418/1.248±0.364	nonionic iso-osmolar contrast media iodixanol		a relative increase in baseline SCr of ≥25% or an absolute increase of ≥0.5 mg/dl (44.2μmol/l) within 48 hours		2.5% (3/118)/3.4% (4/118)
51.87±8.48/53.12±7.55		52 (65%)/47 (58.75%)	1.124±0.128/1.152±0.124	nonionic iso-osmolar contrast medium iodixanol		an increase in SCr of 0.5 mg/dl or 25% above the baseline within 48 hours		2.5% (2/80)/11.3% (9/80)
no		no	1.53±0.44/1.47±0.42	nonionic iso-osmolar contrast medium iodixanol		an increase in SCr more than 0.5mg/dl or more than 25% from the baseline values		2.7% (3/110)/10% (11/110)
75±7/76±7		104 (68%)/92 (60%)	1.20±0.35/1.18±0.33	nonionic iso-osmolar dimeric contrast medium iodixanol		an absolute increase of SCr >0.5 mg/dl within 5 days from the baseline values		9.9% (15/152)/10.5% (16/152)
61.45±8.64/61.44±8.64		963 (64.3%)/991 (66.1%)	1.076±0.259/1.074±0.236	nonionic iso-osmolar contrast medium iodixanol		an increase in SCr≥0.5 mg/dl (44.2μmol/l) or 25% above baseline at 72 hours		2.3% (34/1498)/3.9% (58/1500)
67.5±8.9/67.7±8.9		66 (64%)/76 (73.4%)	1.3±0.4/1.4±0.5	nonionic low-osmolality contrast agent iodixanol		an absolute increase in SCr of ≥0.5 mg/dl or a relative increase of ≥25% measured 48 or 72 hours after the procedure		5.8% (6/103)/8.6% (9/105)
55±6/57±5		35 (53%)/32 (56%)	2.002±0.396/2.002±0.192	no		an increase in SCr by >0.5 mg/dl (44.2μmol/l) or >25% of baseline value		7.7% (5/65)/20% (13/65)

CKD=chronic kidney disease, DM=Diabetes Mellitus, SCr=serum creatinine, NAC=*N*-acetylcysteine. No.=number, E/C=Event(statin)/Control group.

**Table 2:** Quality of included RCTs.

					Blinding				
Study	Jadad score	Allocation concealment	Similarity of baseline characteristics	Eligible criteria	Outcome assessor	Care provider	patients	Completeness of follow-up	Intention- to-treat analysis
Sang-Ho Jo et al. (12)	5	Yes	Yes	Yes	NS	Yes	Yes	Yes	Yes
Maaz Hussian et al. (13)	5	Yes	Yes	Yes	Yes	Yes	Yes	Yes	No
Arezoo Khos et al. (14)	5	NS	Yes	Yes	Yes	Yes	Yes	Yes	No
AnnoToso et al. (15)	5	Yes	Yes	Yes	NS	Yes	Yes	Yes	No
Yaling Han et al. (16)	3	Yes	Yes	Yes	Yes	No	No	Yes	Yes
Okay Abaci et al. (17)	3	NS	Yes	Yes	Yes	No	No	Yes	Yes
Mohamed Shehata et al. (18)	5	NS	Yes	Yes	Yes	Yes	Yes	Yes	No

NS=not specified or available, RCT=randomized controlled trial.
